# Causes of mortality of *Polistes nimpha* colonies

**DOI:** 10.1007/s00040-016-0484-0

**Published:** 2016-03-29

**Authors:** K. B. Kozyra, E. Baraniak

**Affiliations:** Department of Systematic Zoology, Faculty of Biology, Adam Mickiewicz University in Poznań, Umultowska Str. 89, 61-614 Poznań, Poland

**Keywords:** Polistes nimpha, Colonies mortality, Poland

## Abstract

We studied populations of the eusocial paper wasps *Polistes nimpha* from 2012 to 2014, near Poznań, Poland, to identify the causes of mortality and to analyze changes in the number of active nests during the breeding season (from May to September). Results of the 3-year study showed that the major cause of *P. nimpha* mortality (51.9 %) in the study areas was the activity of mammals, probably foxes and wild boars. The second major cause of wasp death was ant predation (12 %). Only 11 of the 308 nests (3.6 %) demonstrated a natural colony decline, following the emergence of reproductives.

*Polistes nimpha* (Christ 1791) build small nests on dead plant stalks, on blades of grass or, less often, on small trees. Colonies are eusocial and are usually haplometrotic (i.e., founded by a single queen) or more rarely, pleometrotic (with two or more queens assisting each other in colony founding) (Blüthgen 1961; Cervo and Turillazzi 1985; Rusina et al. 2007). Haplometrotic colonies are particularly prone to attack by predators and parasitoids until the appearance of the first workers (Strassmann 1981; Makino 1989; Yamane 1996; Rusina 2011; Furuichi 2014). The aim of this study was to determine the major causes of mortality of colonies of *P. nimpha* and to compare the number of nest losses over time.

Observations were conducted from May to September in 2012–2014, within three plots (17.400, 5.300, and 14.200 m^2^) located near Poznań, Poland (52.495705N, 16.874393E; 52.493097N, 16.874016E; 52.495250N, 16.867410E, respectively). Line transects were set out at 2 m apart, and any *P. nimpha* nests occurring along these lines were marked with numbered flags. Nests were observed weekly, and colony development or decline was recorded. The causes of colony death were divided into several categories: (1) nest abandonment by the foundress, (2) mammalian predation, usually only a fragment of the nest pedicel was left on the plant stem where the comb had been attached to it; a lack of nest fragments on the ground indicated that the nest was eaten by a larger mammal, most likely a fox or wild boar or small mammals like rodents. Attacks of birds leave clear traces: torn nest and holes pecked in the comb (Gibo and Metcalf 1978). Such destruction has not been observed by us. For these reason all cases of disappeared combs were interpreted as mammalian predation, (3) ant predation, when observed directly or its traces were visible, i.e., walls of the empty nest had numerous holes made by ants when they were stealing larvae and pupae, (4) anthropogenic factors, if the nest was destroyed as a result of human activity, (5) natural causes, natural colony decline after the emergence of fertile females and males, (6) unknown causes.

A total of 308 nests of *P. nimpha* were observed within the three permanent plots from 2012 to 2014. More than half of the nests were destroyed by mammals (51.9 %). In 84 cases (27.3 %), however, the cause of colony death was unknown (Table 1). Ant predation was detected in 37 colonies (12 %). In 14 cases (4.5 %), nests were abandoned by the foundress, whereas in 11 (3.6 %) colonies, the decline was natural due to the emergence of reproductives. The least significant factor affecting the mortality of paper wasp colonies was human activity, which accounted for less than 1 % (Table 1).

**Table 1:** Types, numbers, and percentages of *Polistes nimpha* colony losses between 2012 and 2014

Year	Mam		Unkn		Ant		Abnd		Nat		Anthr		Total
	*n*	%	*n*	%	*n*	%	*n*	%	*n*	%	n	%	*N*
2012	18	19.1	37	39.4	25	26.6	7	7.4	6	6.4	1	1.1	94
2013	26	45.6	15	26.3	9	15.8	3	5.3	4	7	0	0	57
2014	116	73.9	32	20.4	3	1.9	4	2.5	1	0.6	1	0.6	157
Total	160	51.9	84	27.3	37	12	14	4.5	11	3.6	2	0.6	308

*Mam* predation by mammals, *unkn* unknown cause of colony death, *ant* predation by ants, *abnd* abandoned nests, *nat* natural cause of colony death, *anthr* destruction by anthropogenic factors

A seasonal decline in the number of active nests was observed during each year of the study (Fig. 1). The initial number of nests quickly declined within the first 2–3 weeks of construction. In 2012, 82 % of nests disappeared within the first 2 weeks, compared to 86 % in 2014. This steep decline may be due to the fact that during the first 2 weeks of *P. nimpha* colony establishment, the foundress must prioritize her activities to nest construction and colony development. If she is threatened by a predator, she will abandon her nest construction, which increases her chances of being able to construct another colony or usurp an existing nest nearby (Cervo 2006).

**Fig. 1:**
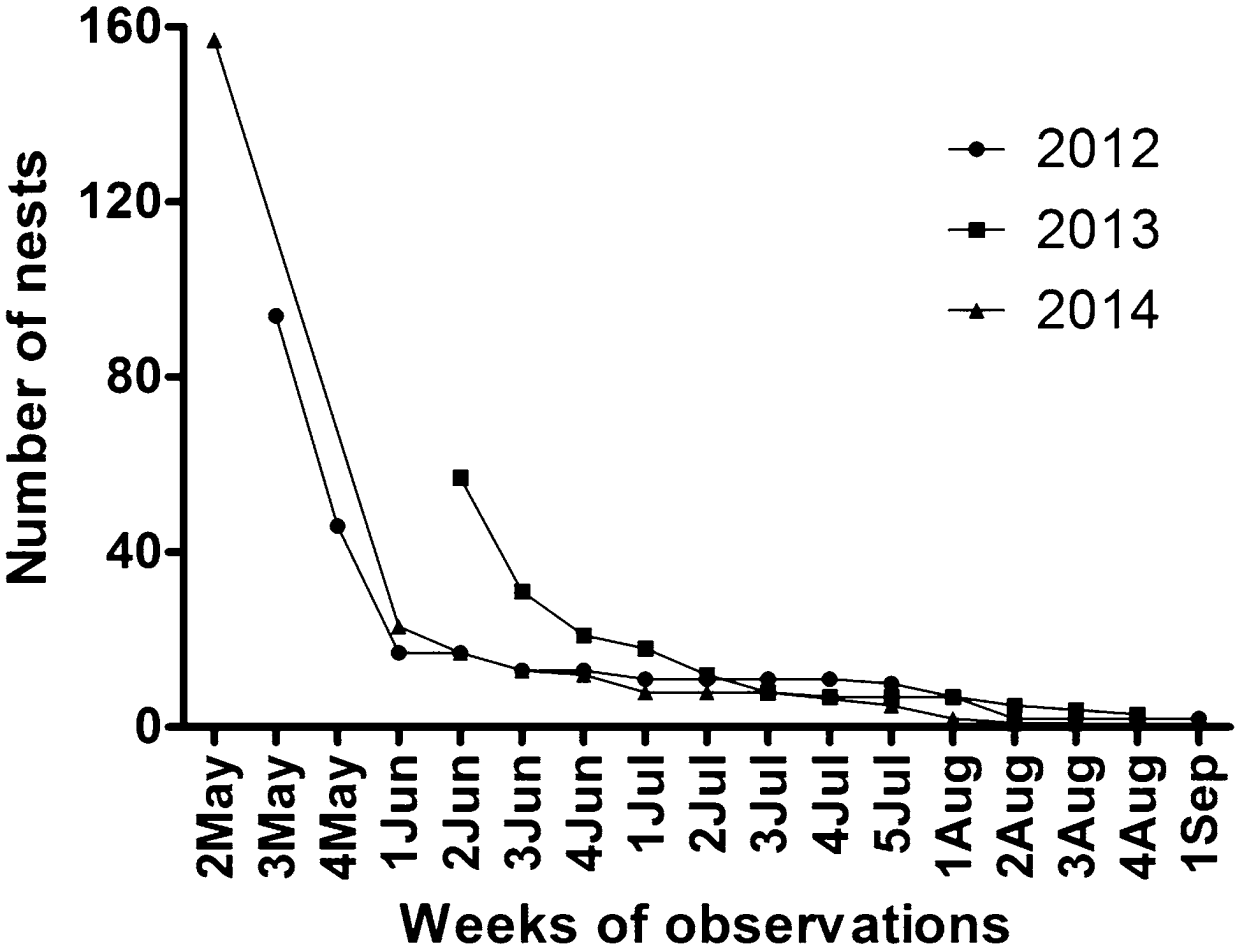
Mortality of *Polistes nimpha* colonies in 2012–2014. Abbreviations: 2May, 3May, etc, successive weeks of months of observations (e.g., *2 May* 2nd week of May)

Following the phase of rapid decline (May to 1st week of June), nest losses lessened. This trend coincided with the appearance of the first workers. In contrast to the queen, worker wasps aggressively defend the nest, making it more difficult to destroy.

Cervo and Turillazzi (1985) reported similar trends in the mortality of *P. nimpha* in Italy, as did Miyano (1980) in *P. chinensis antennalis* in Japan.
